# Investigation of IRES Insertion into the Genome of Recombinant MVA as a Translation Enhancer in the Context of Transcript Decapping

**DOI:** 10.1371/journal.pone.0127978

**Published:** 2015-05-26

**Authors:** Naif Khalaf Alharbi, Senthil K. Chinnakannan, Sarah C. Gilbert, Simon J. Draper

**Affiliations:** 1 The Jenner Institute, University of Oxford, Oxford, OX3 7DQ, United Kingdom; 2 King Abdullah International Medical Research Center, Riyadh, Saudi Arabia; University of British Columbia, CANADA

## Abstract

Recombinant modified vaccinia virus Ankara (MVA) has been used to deliver vaccine candidate antigens against infectious diseases and cancer. MVA is a potent viral vector for inducing high magnitudes of antigen-specific CD8^+^ T cells; however the cellular immune responses to a recombinant antigen in MVA could be further enhanced by increasing transgene expression. Previous reports showed the importance of utilizing an early poxviral promoter for increasing transgene expression and therefore enhancing cellular immune responses. However, the vaccinia D10 decapping enzyme is reported to target and decap vaccinia virus early transcripts – a mechanism that could limit the usefulness of early promoters in MVA viral vectors if this enzyme shows the same activity in this closely related virus. Therefore, we attempted to increase transgene expression in recombinant MVA by inserting the encephalomyocarditis virus (EMCV) internal ribosome entry site (IRES) upstream of a transgene sequence that is controlled by the *B8R* early promoter, and assessed D10 enzyme decapping activity in MVA. The aim of the IRES element was to initiate translation of the transgene transcript (after the removal of the cap structure by the D10 decapping protein) in a cap-independent manner. Here, we report that overexpression of the D10 decapping protein, *in trans*, in MVA reduced growth and transgene expression; however, the IRES element was not able to compensate for the negative effect of the D10 decapping protein. Recombinant MVA with EMCV IRES induced levels of both gene expression and transcription that were similar to the control recombinant MVA, encoding the same transgene but without the IRES element. Both viruses were tested in BALB/c mice and induced similar magnitudes of epitope-specific CD8^+^ T cells. This work indicates that the MVA version of the D10 decapping enzyme, overexpressed using a plasmid, is functional, but its negative effect on transgene expression by recombinant MVA cannot be overcome by the use of the EMCV IRES inserted upstream of the transgene initiation codon.

## Introduction

Modified vaccinia virus Ankara (MVA) has been extensively used in the past two decades to deliver vaccine antigens against many infectious diseases and cancer [1]. Improving the immunogenicity of MVA is desirable [2, 3], and increasing MVA transgene expression is often associated with a higher immunogenicity [4]. Some researchers have reported comparative studies on the use of different strong promoters in recombinant MVA (rMVA) to elicit improved immunogenicity [5]. We have previously reported that utilizing MVA endogenous promoters to drive transgene expression led to a higher magnitude of immunogenicity following rMVA administration [6]. Here, we present our attempt to enhance transgene expression by inserting an internal ribosome entry site (IRES), which is an untranslated, structural RNA element found in many viruses and in some mammalian cells [7], between the ATG start codon of a transgene and a poxviral promoter.

IRES elements initiate translation of the RNA genome of many RNA viruses and mRNA of some DNA viruses, as well as some cellular mRNA, in the complete absence of a 5’7-methylguanylate (m7G) cap [7]. The IRESes can be classified into groups based on their virus family; their requirement for eukaryotic initiation factors (eIFs); or their structural complexity [7, 8]. In the latter, the highly structured IRESes require fewer eIFs. The encephalomyocarditis virus's internal ribosome entry site (EMCV IRES) is always found in the middle of the hierarchy of any of these classification systems [7, 8]. This IRES is neither very complex, nor very unstructured in the structural ranking of IRESes. It is also in the middle of the ranking in terms of eIF requirements as it does not require all eIFs, can function in the absence of eIF4E and eIF4GN, and does not involve ribosome scanning for the ATG start codon as it binds to the ribosome units at the ATG site [7, 8]. EMCV IRES has been shown to be an optimal choice for recombinant protein expression amongst other IRESes [9, 10]. In one report, EMCV IRES was used to drive the expression of uncapped mRNA that was produced by a T7/vaccinia *in vitro* expression system and the recombinant protein expression was enhanced by inserting EMCV IRES between the transgene and the bacteriophage T7 promoter [11].

In the context of RNA capping, poxviruses can produce capped transcripts, but they also decap mRNA to regulate the temporal expression of their genes. Decapping transcripts in poxviruses is mainly due to D10 (expressed late), and to a lesser extent D9 (expressed early), decapping enzymes and they can decap both cellular and viral mRNA in vaccinia virus (VACV) infected cells. Both D9R and D10R genes are highly conserved across the *Poxviridae* family and share 25% sequence similarity [12]. The D10 decapping protein has been well-studied in VACV and shown to have a role in initiating late gene expression, and also has a role in down-regulating early genes by decapping early mRNA that is still present once the D10 enzyme is expressed. This is due to its specificity towards early mRNA as a substrate in general, as it does not appear to preferentially decap specific early transcripts [13]. Previous studies showed that deleting D10 yielded increased early mRNA and delayed late transcription, as well as impairing VACV infectivity and slowing viral growth [12]. Conversely, over-expression of D10 yielded a decrease in the steady-state level of viral late mRNA, decreased protein synthesis, and prevented the formation of infectious virions [12, 13]. These data suggest that specific levels of D10 enzyme are required for optimal VACV growth.

In the case of rMVA vectored vaccines, if the aim is to elicit CD8^+^ T cells, an early promoter is preferred to drive transgene expression because most of the CD8^+^ T cell immunodominant epitopes, from MVA proteins, in humans and mice are products of early genes. In addition, when human dendritic cells were infected with VACV (which is replication-competent unlike MVA), early transcription persisted with no late protein expression [14, 15]. Therefore, insertion of a vaccine transgene downstream of an early promoter is the usual strategy when making a rMVA. However, since early gene transcripts are targeted by the D10 enzyme, we hypothesized that inserting an IRES upstream of a transgene, between the ATG start codon of the transgene and an early promoter, could potentially initiate cap-independent translation of the transgene transcript, thus compensating for the effect of D10 decapping protein. This could subsequently increase the overall expression of the transgene, eliciting improved CD8^+^ T cell responses to the transgenic antigen in vaccinated animals. If decapping is not a prominent mechanism and does not severely affect the transgene transcript, then IRES could still be useful in translating the transgene transcript in a cap-independent manner, allowing its translation to persist (when MVA shifts the expression from early to intermediate and late genes by decapping and other regulatory proteins, in many mammalian cells). Finally it would be informative to assess whether there is any transgene expression increase when two cis-regulatory elements are adjacent to each other; IRES at the RNA level, and promoter at the DNA level. Importantly, IRES has been shown to drive gene expression in the T7/vaccinia expression system despite the effect of an excess amount of D10 enzyme expressed *in trans*, suggesting that IRES could compensate for the negative effects of decapping activity on transgene transcript in poxviruses [13]. Thus the IRES could function in the same way when integrated into a rMVA genome and serve as an optional way to improve MVA-vectored vaccines—in this case, it would also be informative to test the EMCV IRES as translation enhancer with a poxviral promoter and with transcript produced using poxviral transcription machinery. It should be noted that the transcript controlled by the bacteriophage T7 promoter in the study cited above is produced by the bacteriophage T7 RNA polymerase.

Here, we therefore studied the insertion of the EMCV IRES between an early promoter and the ATG start codon of a reporter transgene (rLuc), which is made by the fusion of tPA (tissue plasminogen activator leader sequence that directs the antigen to the secretory pathway of the cell) to pb9 (a strong malaria MHC class I H2-K^d^-restricted epitope from the rodent malaria *Plasmodium berghei* circumsporozoite protein) and then to the *Renilla* luciferase gene. This transgene is under the control of the early *B8R* promoter (pB8), at its natural *B8R* locus. This promoter was selected because the *B8R* gene is fragmented and not essential in MVA [16] and can be replaced with the transgene. Utilizing the pB8 promoter to express the rLuc transgene should result in early transcripts that are suitable substrates for the D10 decapping enzyme; allowing us to assess whether the IRES is then able to drive more expression in a cap-independent manner.

## Results

### The Effect of IRES Insertion on Recombinant Luciferase Expression *in vitro*

**Fig 1:**
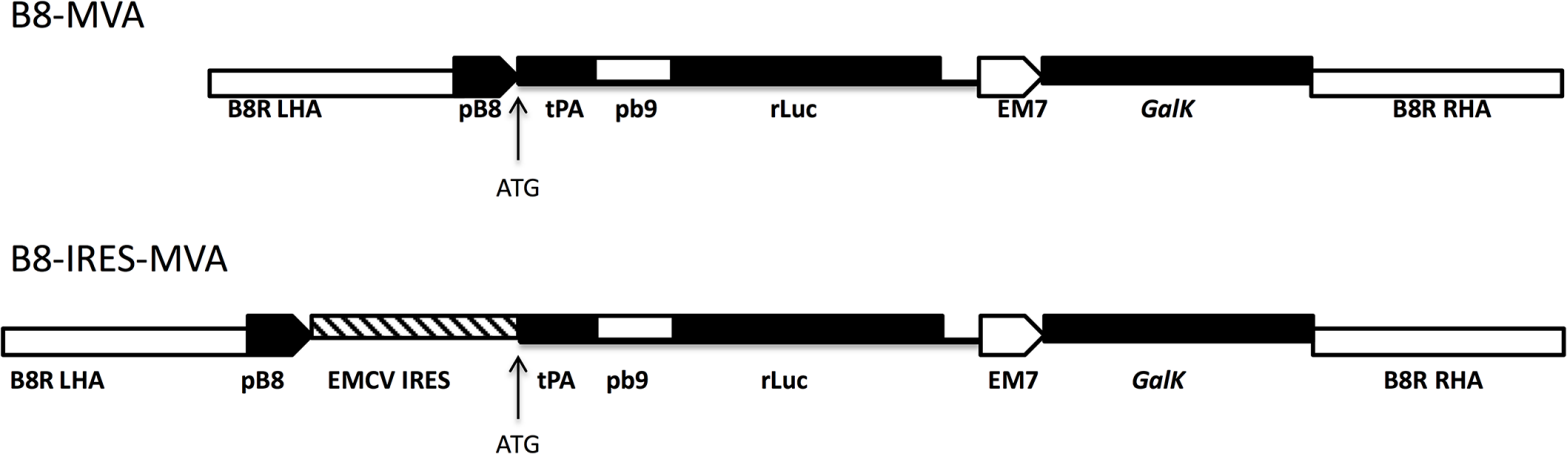
Schematic representation of the rLuc transgene. The rLuc transgene consists of the tPA leader sequence, followed by pb9 (the MHC class I H-2K^d^ epitope of *P*. *berghei* circumsporozoite protein), fused to *Renilla* luciferase (rLuc), under the control of the pB8 promoter and inserted into the *B8R* locus using the *B8R* left homology arm (LHA) and right homology arm (RHA) sequences. It also contains the galactokinase (*GalK*) bacterial selection gene controlled by the prokaryotic EM7 promoter. The transgene is inserted into B8-MVA virus (top), or fused to the encephalitis myocarditis virus’s internal ribosome entry site (EMCV IRES) and inserted into B8-IRES-MVA (bottom). Arrows indicate the used ATG start codons: The ATG start codon of the tPA (Top) and the native 3’ ATG start codon of EMCV IRES (Bottom).

**Fig 2:**
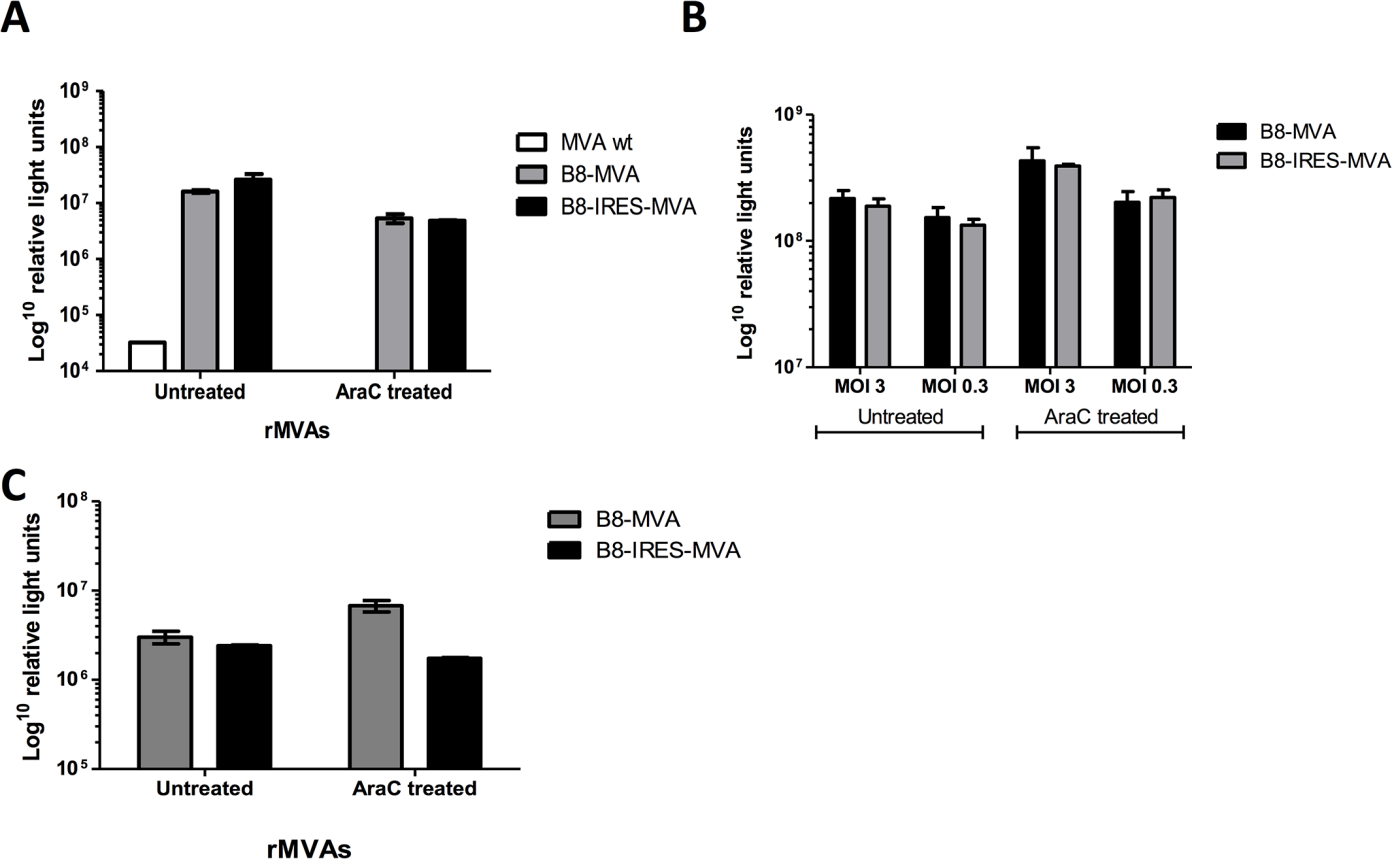
The effect of IRES insertion on luciferase expression of rMVA. (A) BHK-21 (permissive) cells were infected at MOI of 1 with rMVA which either contain the rLuc transgene under the control of B8 endogenous promoter, or the B8 promoter with IRES inserted upstream of the transgene (B8-MVA and B8-IRES-MVA, respectively). Cells were either treated with AraC (to assess the early gene expression) or without AraC treatment (for the overall promoter activity). 24 h.p.i. the cells were lysed, and added to the supernatant and the total level of luciferase expression was measured to determine the effect of IRES insertion on the transgene expression, using the *Renilla* luciferase system. Non-recombinant wild type MVA (MVA*wt*) as a negative control was also included. (B) The same experiment was repeated with different MOI; and **(C)** was also repeated in HEK 293 (non-permissive) cells. All data, shown on a logarithmic scale, represent the mean of 4 wells with SEM error bars. Data are representative of more than five independent experiments.

Two rMVAs were produced, containing the rLuc reporter gene driven by the *B8R* promoter (pB8) at its natural site (Fig 1). One recombinant MVA is named B8-IRES-MVA, and has the EMCV IRES between pB8 and rLuc, the other MVA is without the IRES (named B8-MVA). BHK-21 cells were infected at a multiplicity of infection of 1 (MOI = 1 plaque forming unit, pfu/cell) and incubated at 37°C overnight with or without cytosine arabinoside (AraC), which is used to block DNA replication and prevent late gene expression. After 24 hours, supernatant and cell lysate were collected and pooled together from both AraC treated and untreated cells. Luciferase expression by B8-MVA or B8-IRES-MVA showed no significant difference (Fig 2A). This experiment was repeated thrice independently and showed reproducible results at 24 h.p.i (hours post infection). This experiment was also carried out at an earlier time-point, harvesting the cells at 6 h.p.i. and showed no significant difference between the two rMVAs (not shown due to similarity). The lack of difference was also noted when different MOIs were used; 0.3 and 3 MOI (Fig 2B). Next, there was a concern that BHK-21 (cells permissive to MVA replication) might not be ideal for detecting any expression improvement. So, the HEK 293 cell line (non-permissive to MVA growth) was used and the expression profile was very similar to that achieved with BHK-21 cells (Fig 2C). We had expected to see the effect of the IRES by driving more rLuc expression, especially when the cells were not treated with AraC and late gene expression occurred, which should allow for the expression of the D10 decapping protein. However, our results showed that the IRES insertion did not appear to increase the rLuc transgene expression.

### The Effect of IRES Insertion on the Size of the Recombinant Luciferase Protein

IRES insertion did not yield more luciferase expression, however, we sought to determine whether the IRES could be part of the expressed luciferase protein, and then could have an effect on the size and the secretion of the recombinant luciferase if the translation initiation occurs upstream of the native ATG start codon. Utilizing the native ATG start codon that is found at the 3’ end of the IRES sequence was reported to be important for the IRES efficiency [9]; thus, the rLuc transgene was fused to this native ATG of EMCV IRES when the B8-IRES-MVA was designed. However, IRESes in general have more than one ATG codon, within their sequences, any of which could act as a start codon, and EMCV IRES in particular has ten ATG codons located upstream of its 3’ native start codon [9, 11]. One of these ten ATGs is in the same reading frame as the rLuc transgene ORF in B8-IRES-MVA. If the initiation occurred at this upstream ATG, it would result in a larger rLuc protein in B8-IRES-MVA than in B8-MVA (prior to cleavage of the tPA signal peptide). This larger protein would be 58 kDa compared to the natural start codon-initiated protein, which is a 40.1 kDa protein if the tPA leader sequence is not cleaved or 36.9 kDa after the cleavage of the tPA sequence (as predicted *in silico*). Initiating the translation from an upstream ATG might also interfere with the tPA leader sequence and affect the secretion of rLuc transgenic protein, leading to accumulation and degradation of intracellular rLuc, and presumably to a lower than expected level of expressed luciferase.

**Fig 3:**
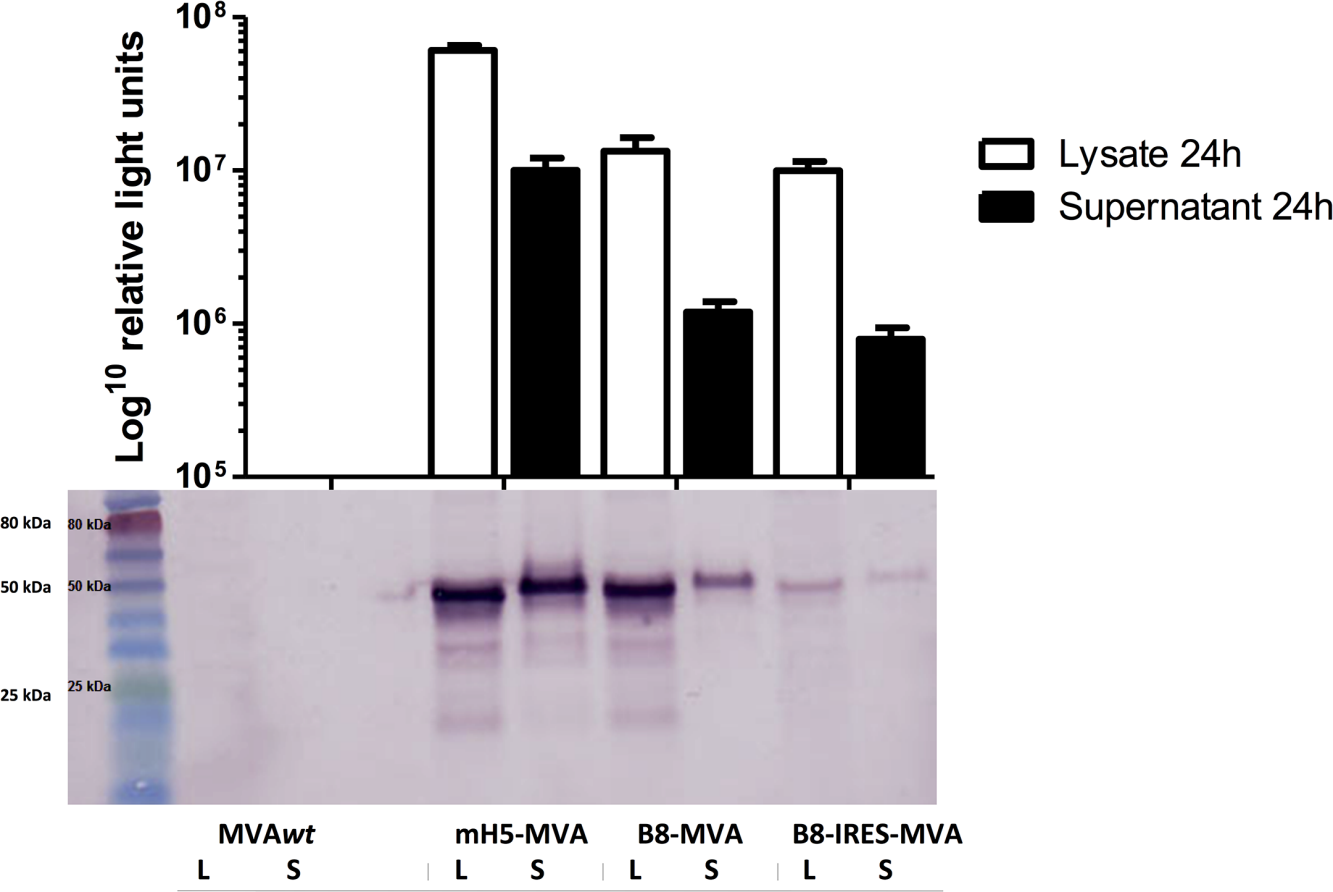
The effect of IRES insertion on secreted luciferase expression. BHK-21 cells were infected with the two rMVAs as in Fig 2 at MOI of 1. 24 h.p.i. the supernatants were collected and the cells were lysed, and the level of luciferase expression in the supernatant or cell lysate was measured to determine the effect of IRES insertion on the secretion and the size of expressed luciferase. The *Renilla* luciferase system, shown on a logarithmic scale, was used (top) and Western blot was also tested (bottom). mH5-MVA as a positive control and non-recombinant wild type MVA (MVA*wt*) as a negative control were also included. The same samples were taken from the same wells for both assays. The data are representative of two independent experiments. L: Lysate from infected cells. S: Supernatant.

A Western blot was therefore performed to determine the size of the luciferase protein expressed by B8-MVA and B8-IRES-MVA recombinants. A rMVA with the modified H5 promoter (mH5) driving the expression of rLuc (mH5-MVA) was included as a positive control for its ability to drive strong luciferase expression [6], and wild-type MVA (MVA*wt*) (empty vector) was used as a negative control. The result showed a similar protein blot for all viruses, in either supernatant or cell lysate samples, with a size of around 50 kDa (Fig 3). Although this size is bigger than predicted, which could be a result of post-translational modifications, it is similar to that of the control viruses, which indicates that the IRES does not impair the secretion of luciferase. Interestingly, the protein abundance seemed relatively similar in supernatant and in cell lysate, especially in mH5-MVA and B8-IRES-MVA (Fig 3), which does not seem in a perfect agreement with the luciferase assay result. In the luciferase assay the level of luciferase expression was higher in cell lysate (around 10-fold) than in supernatant in all tested viruses (Fig 3). This could be because in cell lysate there is a population of immature or degraded luciferase protein that can still emit light when treated with substrate, but cannot bind to the monoclonal anti-rLuc antibody used in the Western blot. However, the Western blot is not quantitative and could also have a lower sensitivity limit compared to the luciferase chemoluminescence assay. Overall, although the IRES insertion did not increase the level of luciferase protein expression, it seemed to have no discernible effect on the size or the secretion of the recombinant luciferase protein.

### The Effect of IRES Insertion on the Luciferase Transcript Level

**Fig 4:**
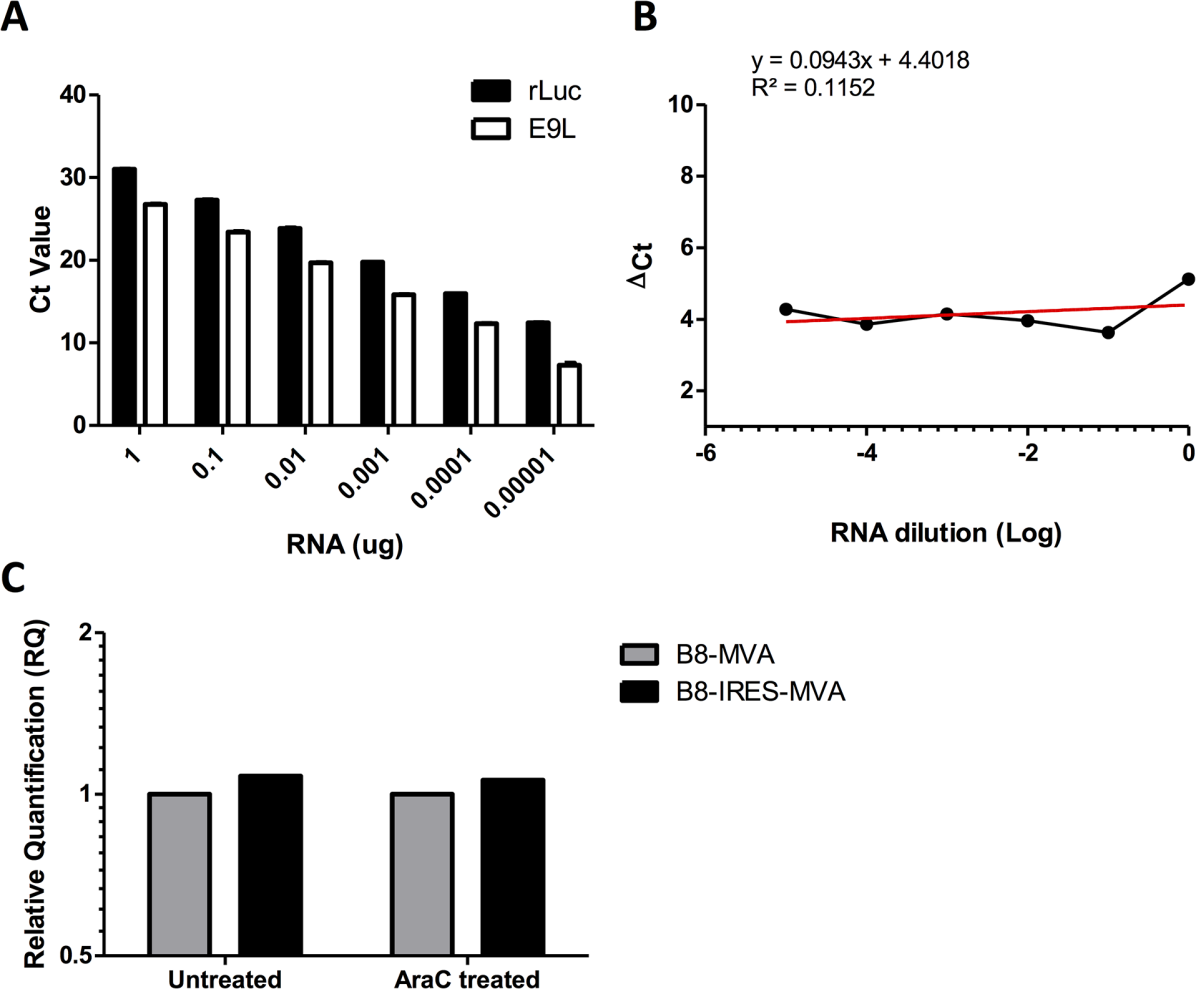
The effect of IRES insertion on luciferase transcript levels in rMVA. BHK-21 cells were infected with the two rMVAs at MOI of 1. Cells were either treated with AraC (to assess the early gene transcription) or without AraC treatment (for the overall transcription). 24 h.p.i. the cells were lysed and the total RNA was extracted and used to make cDNA for the qPCR using the ∆∆ Ct method. This method was validated; (A) the Ct values of E9L and rLuc amplicons are presented against RNA template concentration in μg to show the amplification efficiency of these two genes. (B) The ∆ Ct of every RNA dilution is plotted, with slope of 0.094. (C) The ∆∆ Ct method of qPCR was then performed to determine the relative change fold in the rLuc gene expression. The data are representative of two independent experiments.

There was no observable higher expression of rLuc achieved by the IRES whose role is to increase the translation process; however, any decrease in the rLuc transcript level in B8-IRES-MVA would reduce the protein expression. Moreover, the IRES was inserted between the pB8 promoter and the ATG start codon (Fig 1), resulting in prolonged length of the untranslated region. The untranslated spacer could be crucial for poxviral promoter efficiency according to Di Pilato *et al* [17]. Thus, a comparative ∆∆Ct method of qPCR (a real-time relative quantitative PCR), described by Livak and Schmittgen [18], was used to determine whether the IRES insertion could interfere with the rLuc gene transcription. This method was optimized using the MVA *E9L* gene as an endogenous control to determine the level of luciferase transcript (see Materials and Methods). In the ∆∆Ct method of qPCR, it is critical to have the difference in the Ct values of the tested gene (rLuc) and the endogenous control gene (*E9L*) similar over a serial dilution of a RNA template. These values are then subtracted and the slope of the ∆Ct is plotted. As long as the slope ≤0.1, the amplification efficiency of both *E9L* and rLuc is similar enough and the ∆∆Ct can be calculated to show the relative change in gene expression (Figs 4A and 4B). The result of this assay showed no difference in the relative quantities of luciferase transcript in both viruses, presented as relative fold change in expression (Fig 4C). Inserting IRES did thus not appear to interfere with the rLuc transcript steady-state levels at 24 h.p.i with or without AraC treatment. Interestingly the prolonged untranslated spacer sequence in the rLuc transgene does not seem to have an effect on the pB8 promoter activity.

### *In vivo* Immunogenicity of pb9-rluc Transgenic Antigen

**Fig 5:**
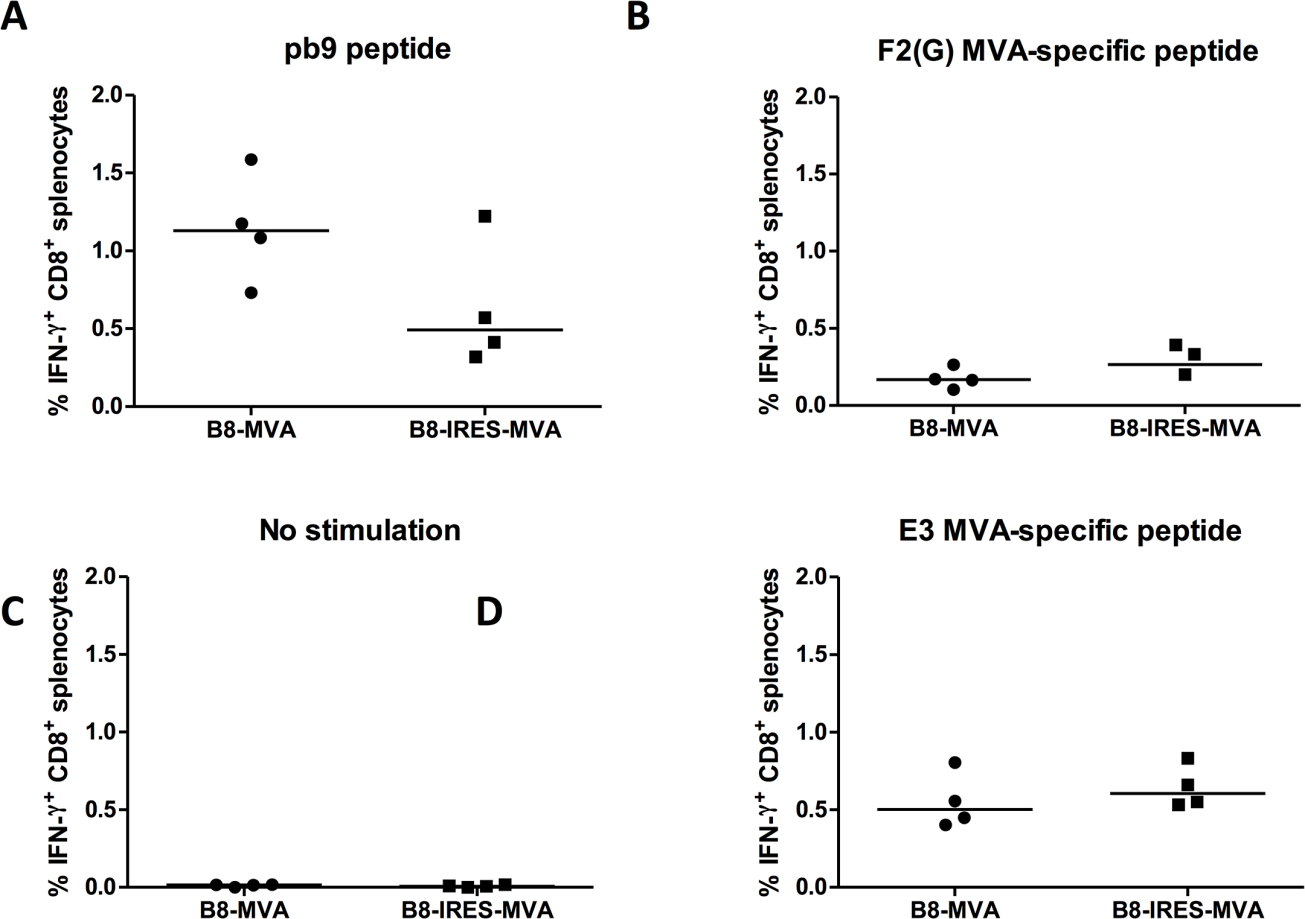
*In vivo* cellular immunogenicity of rMVA with IRES inserted upstream of the rLuc transgene. Two groups of female BALB/c mice (n = 4) were immunized with the respective rMVA. Seven days post-immunization, intracellular cytokine staining and flow cytometry were performed to determine the percentage of IFN-γ-secreting CD8^+^ T splenocytes in response to *in vitro* re-stimulation with (A) pb9 peptide, or (B,D) MVA vector-specific peptides. These values are presented after subtracting the values of (C) unstimulated control cells for every mouse sample. The median of each group is shown. Data are representative of two independent experiments.

The ultimate aim of the IRES insertion study was to improve the MVA vaccine vector by obtaining increased transgene expression, which could result in higher immunogenicity. Although stronger expression was not observed using IRES *in vitro*, we continued the study by performing a small *in vivo* immunogenicity experiment in mice in order to confirm whether the use of the IRES *in vivo* would mirror the findings in cell lines *in vitro*. Two groups of four BALB/c mice were immunized with B8-MVA or B8-IRES-MVA as described in the materials and methods. The percentage of antigen-specific IFN-γ producing CD8^+^ splenic T cells was determined, seven days after a single shot of rMVA, by intracellular cytokine staining and flow cytometry. The responses to the transgenic peptide (pb9) or to the MVA vector-specific F2(G) and E3 peptides were similar between the groups (Fig 5). This experiment was performed twice independently and showed similar results. In fact, the B8-IRES-MVA induced a lower pb9-specific response. This result showed that IRES insertion into the rMVA genome did not yield higher cellular immune responses, consistent with the analyses of transgene expression *in vitro* using cell lines and Western blotting, suggesting this assay presents antigen levels that are reflective of *in vivo* outcome.

### IRES Functionality in the Presence of the D10 Decapping Protein

**Fig 6:**
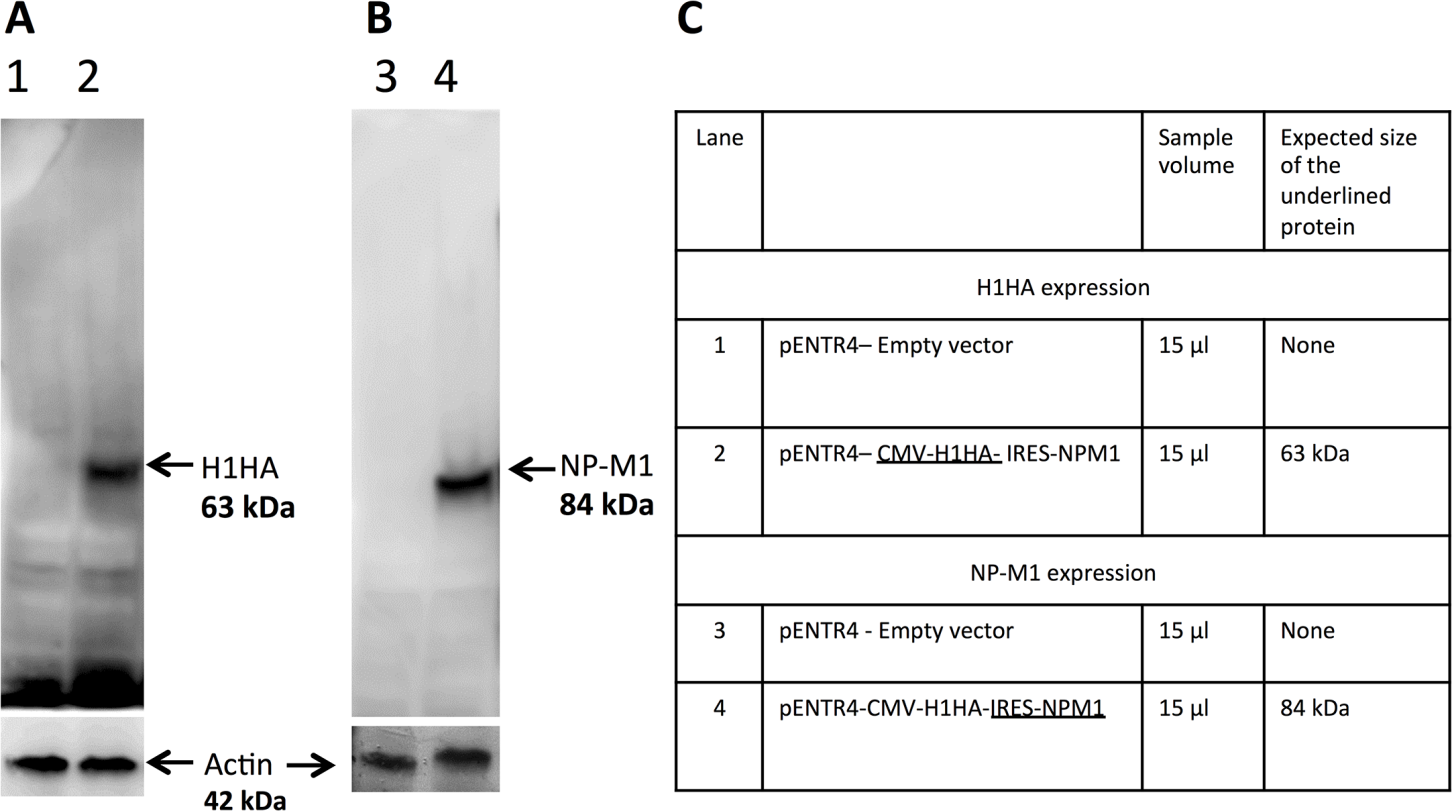
IRES functionality in a bicistronic expression system. HEK 293 cells were transfected with a bicistronic vector expressing Influenza H1HA antigen under the control of a CMV promoter, and the Influenza NP-M1 fused antigen under the control of EMCV IRES. (A) H1HA was detected by Western blot (lane 2) using anti-HA antibody. (B) NP-M1 was detected by Western blot (lane 4) using anti-M antibody. Lane 1 and 3 contained cell lysates that were transfected with empty vector. Actin was detected as a control in all lysates. (C) A table explaining the expression and the expected size of the proteins.

The EMCV IRES has not (to our knowledge) been reported as non-functional, in particular when used in bicistronic expression vectors, which allow the simultaneous expression of two proteins separately but from the same RNA transcript [9]. We therefore also assessed the IRES functionality in a bicistronic expression system by performing a Western blot on HEK 293 cell lysate transfected with a bicistronic vector expressing Influenza virus heamagglutinin 1 antigen (H1HA) under the control of a CMV promoter [19], fused to another antigen of Influenza, nuclear protein and matrix 1 protein (NP-M1), under the control of the EMCV IRES (identical to the one used in B8-IRES-MVA). The EMCV IRES was clearly able to express the Influenza NP-M1 fused antigen, with the expected size of 84 kDa (Fig 6). This confirmed the functionality of the EMCV IRES sequence used here in driving cap-independent expression.

**Fig 7:**
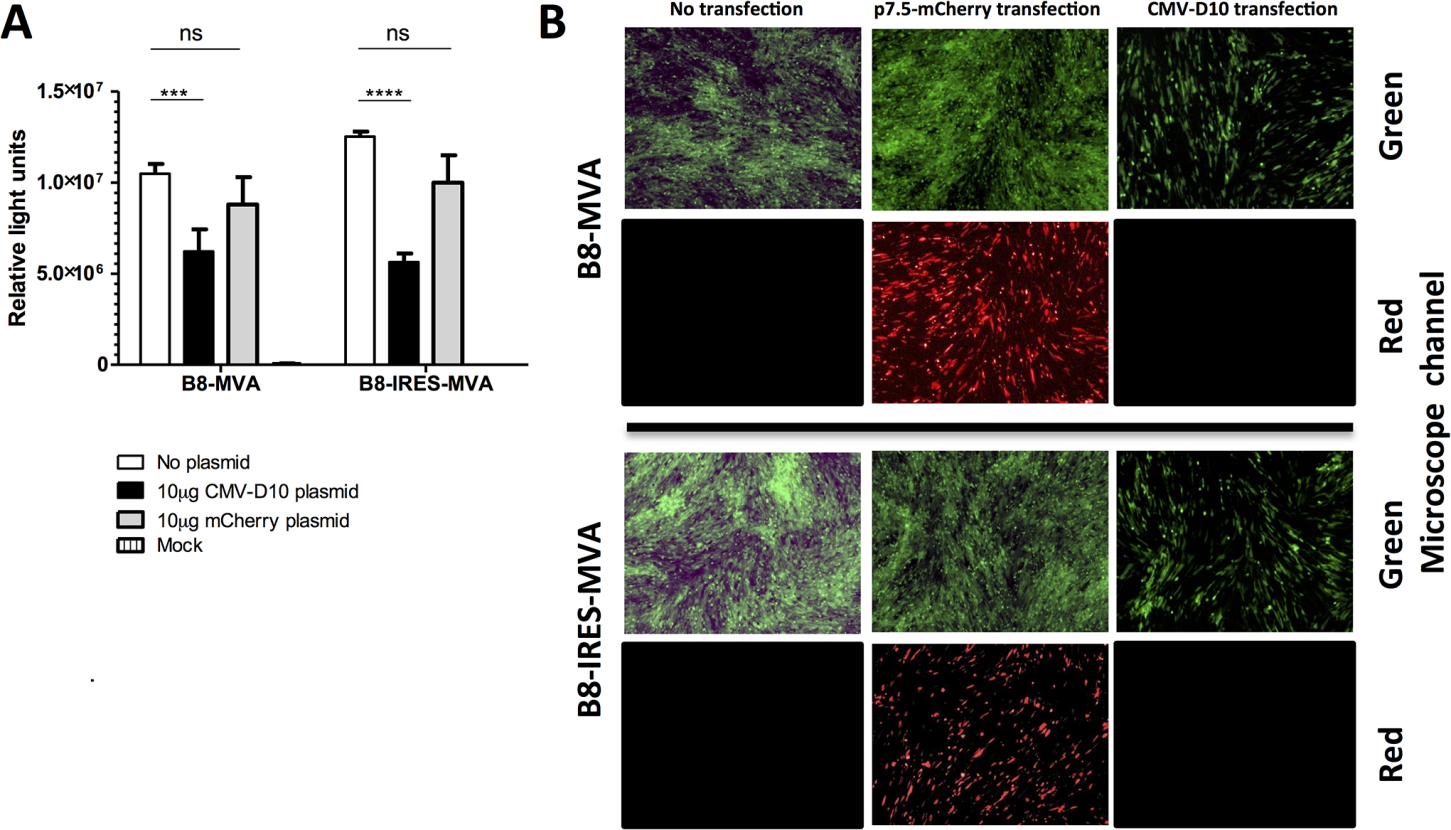
The effect of IRES insertion on luciferase expression by rMVA in the presence of overexpressed D10 decapping protein. (A) BHK-21 cells were transfected with CMV-D10 plasmid, p7.5-mCherry plasmid (irrelevant transfection), or left untransfected (no plasmid). 12 h later cells were infected with either B8-MVA or B8-IRES-MVA at MOI of 1. 24 h.p.i. the cells were lysed, and added to the supernatant and the total level of luciferase expression was measured using the *Renilla* luciferase system to determine the effect of IRES insertion on the transgene expression in the presence of an excess amount of D10 decaping protein. The data represent the mean of 4 wells with SEM error bars and are representative of two independent experiments. ***, *P* = 0.001. ****, *P* < 0.0001. ns, not significant by two-way ANOVA with Bonferroni post-test. (B) The cells were imaged to show the effect of transfection with D10-expressing vector or the irrelevant plasmid (expressing mCherry) on GFP expression—which reflects the viral growth.

It remained possible, however, that the IRES would not function when used between the transgene and the promoter, integrated into the recombinant B8-IRES-MVA. Therefore, we sought to test its functionality in the context of decapping activity. Here, the *D10R* gene, encoding the main decapping enzyme (D10 protein), amplified from the MVA genome, was cloned downstream of a CMV promoter in a mammalian cell expression plasmid, named CMV-D10. This was used to overexpress D10 protein to determine whether the IRES would function in the tested cell line. This experiment should also reveal whether the decapping actually occurs as a result of the MVA D10 protein. We noticed that the MVA D10 enzyme has 3 amino acid substitutions (due to point mutations), which are absent in VACV (S1 Fig). Notably, the VACV D10 activity was shown to be inhibited by induced point mutations [20]. However, the reported point mutations in the VACV D10 protein were in the active motif whereas those we found in the MVA D10 protein were not, and may not have a functional effect on the MVA D10 (S1 Fig). Therefore, to test the functionality of the EMCV IRES in the presence of an excess amount of MVA D10 protein, monolayers of BHK-21 cells in 6-well plates were transfected with 10 μg of CMV-D10, or irrelevant plasmid expressing mCherry gene (red fluorescent marker) driven by the poxviral p7.5 promoter, or left with no transfection. Twelve hours later, cells were infected with either B8-MVA or B8-IRES-MVA at a MOI of 1, or left uninfected. The untransfected cells were also infected with either of the viruses to serve as controls. Pools of lysate and supernatant were collected 24 h.p.i. The luciferase level was reduced by CMV-D10 treatment in the cells infected with either B8-MVA or B8-IRES-MVA as compared to the control (untransfected, but infected cells, Fig 7A). In contrast, the level of luciferase in cells treated with mCherry plasmid did not drop in both viruses, which suggested that the reduction in the luciferase level is specifically due to the D10 treatment. However, the presence of the IRES did not improve the rLuc expression in a cap-independent manner as hypothesized. Therefore, we concluded that the MVA version of D10 decapping protein is active when overexpressed *in trans*, but the EMCV IRES could neither initiate cap-independent translation of the transgene transcript, nor compensate for the D10 negative effects as originally hypothesized.

### D10 decapping activity reduces MVA viral growth

The MVA D10 protein, which was shown to be active in the previous experiment, has been reported to act on the majority of capped cellular and poxviral mRNA (regardless of what these mRNA molecules encode), and is efficient in decapping RNA of 24–309 nucleotides in length [21]. Although the D10 enzyme does not have a clear specificity, there is more chance of decapping cellular and early poxviral mRNA as opposed to intermediate and late poxviral transcripts [21]. Therefore, to determine if the D10 overexpression had a deleterious effect on MVA growth by decapping, we imaged the cells that had been transfected with CMV-D10 and infected with rMVAs (used for the IRES functionality experiment above). At 24 h.p.i. it was clear from the fluorescent microscopy that the D10 treatment reduced the green fluorescent protein (GFP), which is linked to the viral growth (given the GFP gene is also inserted elsewhere into the rMVA genome) (Fig 7B). This suggested that the overexpression of MVA D10 protein did reduce the viral growth despite the three amino acid differences in comparison to VACV. This reduction was unlikely due to the transfection process, as the irrelevant transfection with mCherry-expressing vector did not reduce the viral growth, whilst the mCherry red fluorescence was also detected confirming successful transfection in the control. Because the mCherry gene is driven by a poxviral promoter, its expression would only occur in cells that are both infected and transfected. Overall these data suggest the MVA version of D10 decapping enzyme is functional, when it was overexpressed using a plasmid, and was able to reduce transgene expression as well as viral growth.

## Discussion

We attempted to enhance rMVA immunogenicity by increasing transgene expression, through insertion of an IRES between the ATG start codon of the transgene and the pB8 promoter, aiming to overcome the negative effects of decapping activity on the transgene. IRES elements have been used in bicistronic constructs integrated into VACV genome, in previous studies [22]. Here, the EMCV IRES was tested first in a bicistronic expression system and proved functional in expressing influenza virus proteins in a cap-independent expression. Thus, the same EMCV IRES sequence was used to try and increase the expression of luciferase in rMVA.

Initially we generated two rMVA vectors encoding the rLuc transgene at the *B8R* locus. In one vector the IRES was inserted between the rLuc transgene and the pB8 promoter, which is an early promoter leading to an early transcript that should be a suitable substrate for the D10 decapping enzyme. We initially tested these viruses *in vitro*, where the D10 protein should be expressed late in the MVA life cycle, i.e. when cells were not treated with AraC (an agent used to block DNA replication and study the early gene expression in MVA); and the IRES could also potentially help by increasing the luciferase expression, at least, in the untreated cells. However, IRES insertion did not seem to protect the tPA-pb9-rLuc transcript from any negative effects of decapping by measuring luciferase expression in BHK-21 (permissive to MVA) or HEK 293 cells (non-permissive) in the presence or absence of AraC treatment. Western blotting showed that the protein expressed by either B8-MVA or B8-IRES-MVA virus is similar and around 50 kDa, suggesting that the IRES insertion did not affect the translational ATG start codon. It also showed that the IRES did not appear to interfere with the luciferase secretion, suggesting that the tPA leader sequence was not affected by the upstream inserted IRES sequence. Relative qPCR also showed that the IRES inserted between the promoter and the transgene ATG start codon (thus elongating the untranslated spacer), did not affect the pB8 promoter activity. This contradicts a previous report emphasizing the importance of the spacer region for poxviral promoter activity [17]. Finally, the IRES did not appear to improve the cellular *in vivo* immunogenicity when the rMVAs were tested in mice, presumably because it could not drive more transgene expression in the mouse model, confirming the results from the cell lines as tested by Western blot. Unlike the luciferase enzymatic assay, the Western blot showed lower levels of luciferase in the B8-IRES-MVA infected lysates, which was associated with a lower pb9-specific response in mice. This suggests that the protein levels rather than the enzymatic levels might be the suitable correlate to predict *in vivo* immunogenicity. Nevertheless, these data led us to conclude that either the IRES is somehow impaired when placed between the transgene and the promoter and inserted into the rMVA genome, or that decapping is not a very prominent mechanism in MVA; at least in this experimental setting.

The optimal sequence of the EMCV IRES should have a number of characteristics to ensure efficient expression, according to Bochkov and Palmenberg [9]. First, the EMCV IRES should start at the preferred 5’ boundaries of EMCV IRES and end at the 3’ minimum boundaries of EMCV IRES (highlighted in S2 Fig). Second, it should utilize the defined ATG start codon at the 3’ end of the IRES, and not any of the other upstream ATG codons within the IRES sequence. Third, it should contain the A6 loop (6 consecutive residues of adenine), which is the native and optimal sequence, and not the A7 loop (7 consecutive residues of adenine), which was introduced in some EMCV IRES versions to slightly reduce the IRES-controlled expression for specific applications (see [9]). Therefore despite the fact that the EMCV IRES functioned efficiently in our bicistronic expression system, we also confirmed that the EMCV IRES sequence used here is the optimal sequence (S2 Fig), by performing DNA sequencing on viral DNA from B8-IRES-MVA.

Bochkov and Palmenberg had also warned that placing the IRES immediately next to the 5’ cap, i.e. between the ORF and its promoter, in monocistronic expression vectors might cause the IRES to fail or interfere with the translation process. The IRES in our B8-IRES-MVA is slightly mimicking the monocistronic systems and we detected slightly lower protein levels as tested by Western blot (although that was not the case in the qPCR and luciferase assay). However, it remains possible that the IRES RNA did not fold into a functional structure when it was placed too close to the pB8 promoter in our case, and a spacer sequence could be tested between the IRES and the promoter in any future studies. A similar situation was previously noted when the EMCV IRES was inserted into the VACV genome downstream of the T7 bacteriophage promoter. The IRES did not efficiently translate the uncapped transcript provided by the T7/vaccinia system until the T7 structured loop was inserted between the T7 promoter and the EMCV IRES, which obviously acted as a spacer [11]. We have not tested whether a structured loop (a hairpin structure) could improve the efficiency of the EMCV IRES when used with a poxviral promoter and this should be open for future research. In addition, it is likely that the transgene transcript, in our study, is capped despite the presence of the IRES RNA so the ribosomes could be attracted to its cap structure rather than the IRES. It is worth noting here that the IRES was used in the T7/vaccinia expression system mainly because this system produces a large amount of uncapped mRNA.

Nevertheless, we continued to assess the IRES functionality in the context of decapping. An over-expression system was set up by transfecting BHK-21 cells with a D10-mammalian expression plasmid prior to infecting with the rMVAs. This system aimed to show the role of the EMCV IRES in expressing the early transcripts when the D10 decapping protein (acting on these early transcripts) is over-expressed. However, the IRES in B8-IRES-MVA did not increase the level of luciferase compared to the control (B8-MVA). In D10-transfected cells, both B8-MVA and B8-IRES-MVA showed a lower level of luciferase than the controls (B8-MVA and B8-IRES-MVA without D10 transfection). This suggested that the D10 decapping enzyme is active in MVA, when overexpressed using a plasmid, and could affect the transgene transcription, but that the rLuc transcript was not translated in a cap-independent manner by inclusion of the IRES as originally hypothesized. Decapping enzymes in poxviruses are well studied in VACV and reported to have very conserved genes across the *Poxviridae* family. Here we studied the D10 decapping enzyme in MVA rather than in VACV. Interestingly, an excess amount of the D10 protein reduced the viral growth as compared to the irrelative transfection of mCherry marker. This supports the previous studies [13], carried out on VACV, that the over-expressed D10 decapping enzyme targets early mRNA transcripts and impairs the viral growth. Although other groups [20] have reported that individual point mutations in the active motif of D10 could inhibit its activity in VACV, we report here that the MVA D10 protein appeared to function in spite of having three amino acid differences, but not in the active motif as compared to VACV D10 protein.

Overall, our data suggest that inserting the EMCV IRES between a poxviral promoter and the ATG of a transgene, integrated into the MVA genome, does not increase the transgene expression or the *in vivo* cellular immunogenicity.

## Materials and Methods

### Ethics Statement

All animal procedures were performed in accordance with the terms of the UK Animals (Scientific Procedures) Act (ASPA) for the Project Licence (PPL 30/2889) and were approved by the University of Oxford Animal Care and Ethical Review Committee. All mice were housed at least 7 days for settlement prior to any procedure in the University animal facility, Oxford, UK under Specific Pathogen Free (SPF) conditions.

### The transgene construct

The rLuc transgene was previously described [6]. Briefly, a cDNA encoding a variant of *Renilla reniformis* (sea pansy) luciferase, rLuc, which exhibits improved stability and light output [23], was obtained from Dr Sanjiv Gambhir, Stanford University, USA. A poxviral early transcription termination motif (T5NT) was removed by PCR mutagenesis, such that the isoleucine at position 48 is encoded by ATC instead of ATT. We further modified the encoded protein by fusing two sequences to the N-terminus: the H2-K^d^ restricted murine CD8^+^ T cell epitope SYIPSAEKI (pb9) from the *Plasmodium berghei* circumsporozoite protein [24] and the signal peptide comprising amino acids 1–28 of human tissue plasminogen activator (tPA). The sequence MDD linked tPA and pb9 and the sequence GS linked pb9 and rLuc. A T5NT early termination sequence was placed immediately downstream of the tPA-pb9-rLuc open reading frame. The resulting construct, tPA-pb9-rLuc, encodes a secretable, pb9-tagged version of rLuc, with enhanced extracellular stability [23], and suitable for poxviral early expression.

### Transgene insertion into *B8R* locus using MVA-BAC

Construction and generation of MVA-BAC and generation of MVA deletion mutants using *GalK* recombineering [25], has been described previously [26]. We employed this method to generate recombinant MVA (rMVA) viruses expressing the rLuc transgene under the control of the pB8 promoter at the natural *B8R* locus. A cassette was constructed using conventional PCR and restriction enzyme based cloning, comprising the bacterial *GalK* resistance gene and the rLuc transgene (tPA-pb9-rLuc) with or without EMCV IRES (Fig 1). This was amplified with Phusion (Finnzymes) as one insert (i.e. a targeting DNA) for recombineering by using long oligonucleotide primers (Eurofins MWG Operon) to add 50 bp homology arms to the 5’ and 3’ ends. The primers were designed to delete the viral *B8R* ORF wholly and to replace it with the rLuc transgene insert, which also contained the *GalK* bacterial selectable marker. The homology arm immediately 5’ to the tPA-pb9-rLuc ORF was designed to place the initiator codon (ATG) of the inserted transgene at the same position as that of the deleted viral *B8R* gene. These targeting constructs (Fig 1) were confirmed by DNA sequencing and used for MVA-BAC recombineering as previously described [26]. *GalK* selection was used to facilitate removal of the marker and ‘recycling’ for insertion at a second locus, though we did not take advantage of this in the present study.

### Cells, MVA-BAC rescue, and purification of rMVA

The recombineered MVA-BACs were rescued to recombinant MVA in BHK-21 cells (obtained from ATCC via LGC Standards) using a fowlpox virus helper as previously described [26]. To avoid a second round of recombineering, and to establish viral viability at an early stage, the *GalK* gene was not removed prior to rescue. BACs and derived viruses were checked for identity and purity by PCR and the sequences of the homology arms and transgenes were confirmed at both stages. BAC-derived rMVAs were plaque-picked three times to ensure purity, as a precautionary measure: carry-over of *GalK*-negative ‘‘hitch-hikers” is sometimes problematic in this positive metabolic selection system (this can alternatively, or additionally, be addressed by repeated bacterial re-streaking on MacConkey indicator plates [27]). The viruses were amplified in 1500 cm^2^ monolayers of BHK-21 cells, partially purified over sucrose cushions and titred in BHK-21 cells according to standard practice, and purity and identity were again verified by PCR. Since MVA-BAC has a GFP marker gene under control of the Fowlpox virus p4B promoter [26], all the rMVAs expressed GFP in addition to the rLuc transgenic antigen.

### Luciferase assay

For luciferase assays, a ‘‘spinoculation” protocol was used [28] in order to synchronize the infection and enable prior washing of the cells to remove rLuc activity in the inoculum. BHK-21 cells (10^6^ cells/well) in flat-bottom 6-well plates were inoculated in duplicate with rMVAs at 1 pfu/cell. The plates were centrifuged at 650 x *g* for 1 h at 0°C then washed three times with ice-cold DMEM containing 2% FCS, before being placed at 37°C in 1 mL per well of medium which optionally contained 40 mM cytosine arabinoside (AraC). The supernatants were collected at 24 h post-infection, and the cells were washed in PBS and lysed in a volume of 1 mL. The rLuc activity in 10 μL aliquots of these samples was quantified using the *Renilla* Luciferase Assay System (Promega) and a Varioskan Flash luminometer (Thermo).

### Western blot

Western blot was performed according to standard practice and using 12% Precise Tris-Glycine Gels (Thermo Scientific) and Trans-Blot Turbo Mini PVDF Transfer Packs (Bio-Rad). Mouse anti-*Renilla* luciferase antibody, clone 5B11.2 (Millipore) was used, in 1:1000 dilution, to detect the rLuc transgenic antigen. Donkey anti-mouse IgG conjugated to alkaline phosphatase and SIGMAFAST BCIP/NBT tablet (Sigma-Aldrich) were used to develop the immune reaction. The molecular weights of proteins were compared to the ColorPlus Protein Molecular Weight Markers (New England Biolabs). For H1HA blots, rabbit anti H1HA (diluted 1:500, Sigma: SAB3500059) was used as primary antibody with HRP-conjugate anti-rabbit (diluted 1:5000, Alpha diagnostics, Cat# 20120) as a secondary antibody. For NP-M1 blots, the primary antibody was mouse anti M1 (diluted 1:250, Abcam, Cat# 22396) and the secondary antibody was HRP-conjugate anti-mouse (diluted 1:2500, Alpha diagnostics). Primary rabbit anti-Actin (diluted 1:2500, Sigma: A2066) with secondary anti-rabbit-HRP (diluted 1:5000, Alpha diagnostics: Cat# 20120) were used for Actin blots.

### RNA extraction, cDNA synthesis, and real-time relative quantitative PCR

rMVA were used to infect monolayers of BHK-21 cells in 6-well plates at MOI of 1 and, 24 h.p.i., the supernatants were then removed and cells were washed with PBS and harvested by scraping. The cell lysates were spun at 18,000 x *g* for 1 min, then the pellets were used to extract the total RNA using the RNeasy Mini Kit (QIAGEN), and 1 μg of total RNA was used to synthesize cDNA using the Omniscript RT Kit (QIAGEN). The qPCR reaction was then set up to amplify rLuc gene (test) and *E9L* gene (endogenous control) using the primer pairs: q-rLuc-F ccctgatcaagagcgaagag, q-rLuc-R gtctaacctcgcccttctcc, q-E9-F gagtatagagcactatttctaaatccca, and q-E9-R tcaactgaaaaggccatctatga. Two wells in the 96-well qPCR plate were used for every sample (or a dilution of a sample) to detect either rLuc or *E9L*, because specific labelled probes to detect those genes were not used. Thus, the cDNA samples, each made from 1 μg of total RNA, were used with the SYBR green master mix (QuantiTect SYBR Green PCR Kit, QIAGEN) following this thermocycling program: 95°C for 10 min, followed by 40 cycles of 95°C for 10 s and 60°C for 40 s. All kits were used as instructed by the manufacturers. The real-time relative quantitative PCR was performed using Step One Plus thermocycler (AB Applied Biosystem) and the results were analysed using the ΔΔ Ct method as described previously [18].

### *In vivo* immunogenicity, ICS, and flow cytometry

Female BALB/c mice (Harlan, UK) aged 6 to 8 weeks were immunized intramuscularly (i.m.) in the tibialis muscles (total volume 50 μL) with a total of 10^6^ pfu of rMVA. Mice were used in accordance with the UK Animals (Scientific Procedures) Act 1986 under project licence number PPL 30/2889 granted by the UK Home Office. For induction of short-term anaesthesia, animals were anaesthetised using vaporised IsoFloH. Splenocytes were harvested seven days post-immunization for analysis by flow cytometry with intracellular cytokine staining (ICS), both as previously described [19], using re-stimulation with 1 μg/mL pb9, F2(G), or E3 peptides [29]. In the absence of peptide restimulation, the frequency of IFN-γ^+^ CD8^+^ cells was 0.1% by flow cytometry. This frequency was subtracted from tested restimulated samples.

### Statistical analysis

GraphPad Prism (GraphPad software) was used for statistical analysis and to plot data.

## Supporting Information

S1 Fig Alignment of the vaccinia virus D10 peptide sequence. Alignment of the D10 protein sequences in the vaccinia virus Western Reserve strain (VACV WR) and in MVA. The three amino acid substitutions are in italicised letters with lack of stars underneath. Bold letters indicate the active motif of the D10 enzyme (22 amino acids, residues 122 to 149). GenBank accession numbers for D10 protein: AAB96518 (for MVA) and YP_232997 (for VACV-WR).
(TIFF)

S2 Fig EMCV IRES nucleotide sequence. The 557 nucleotide sequence of the EMCV IRES (GenBank Accession: KF836387.1), used in this study to design the B8-IRES-MVA, was confirmed by DNA sequencing, presented here. The optimal EMCV IRES sequence according to Bochkov and Palmenberg [9] should have the A6 loop (boxed) and utilizes the defined native 3’ ATG start codon (in shaded box), not any of the upstream non-defined ATG (in bold). The minimum and preferred boundaries of the EMCV IRES are labelled with arrows. The IRES encompasses the minimum boundaries at both ends, but with the preferred boundary at the 5’ end.
(TIFF)
