# Supplementary figures and images for: Investigation of IRES Insertion into the Genome of Recombinant MVA as a Translation Enhancer in the Context of Transcript Decapping
Source: PLoS One. 2015 May 26;10(5):e0127978. doi: 10.1371/journal.pone.0127978 (PMC4444188; doi:10.1371/journal.pone.0127978)

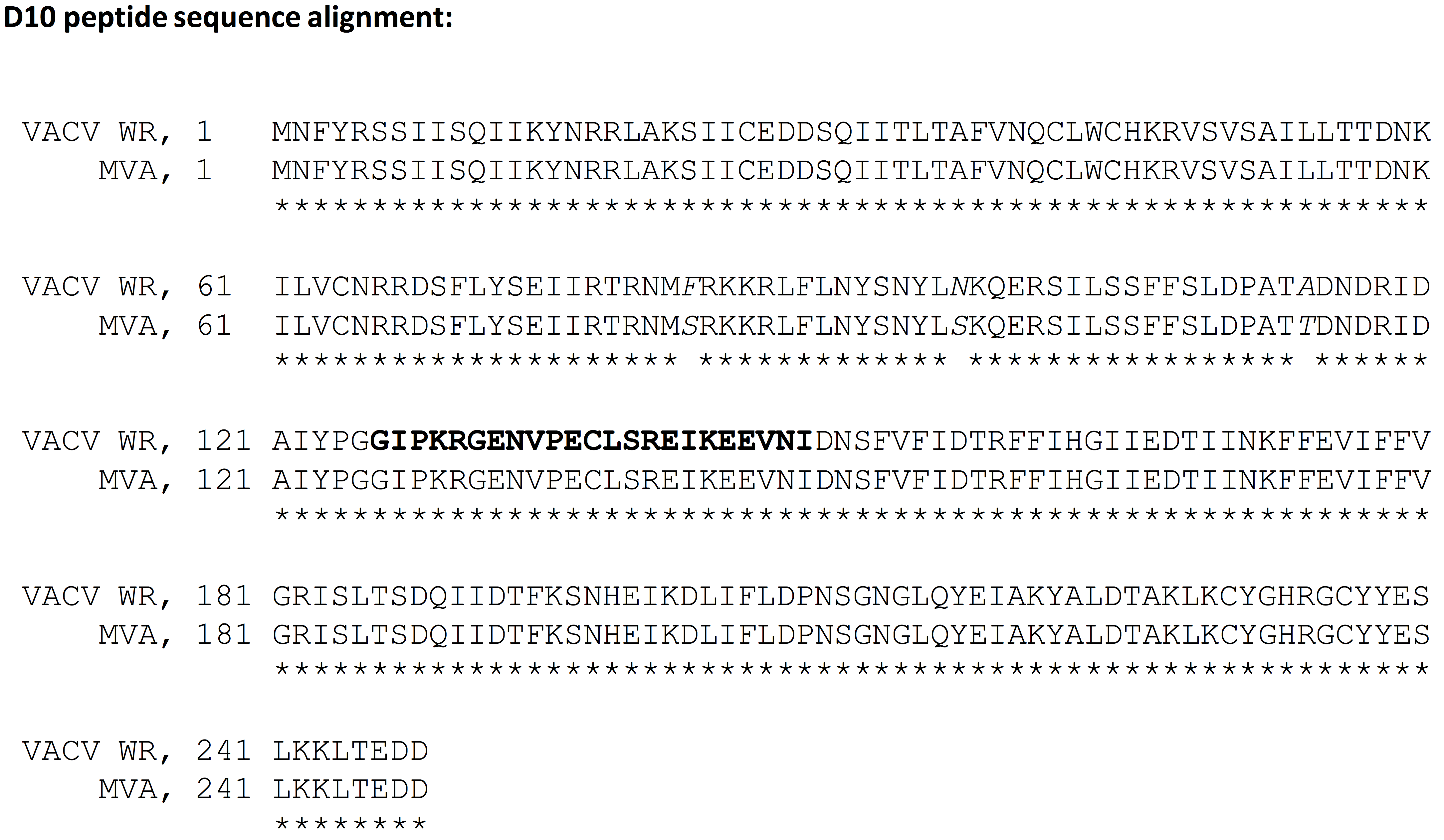

Supplement: S1 Fig — Alignment of the D10 protein sequences in the vaccinia virus Western Reserve strain (VACV WR) and in MVA. The three amino acid substitutions are in italicised letters with lack of stars underneath. Bold letters indicate the active motif of the D10 enzyme (22 amino acids, residues 122 to 149). GenBank accession numbers for D10 protein: AAB96518 (for MVA) and YP_232997 (for VACV-WR). (TIFF) [file pone.0127978.s001.tiff]

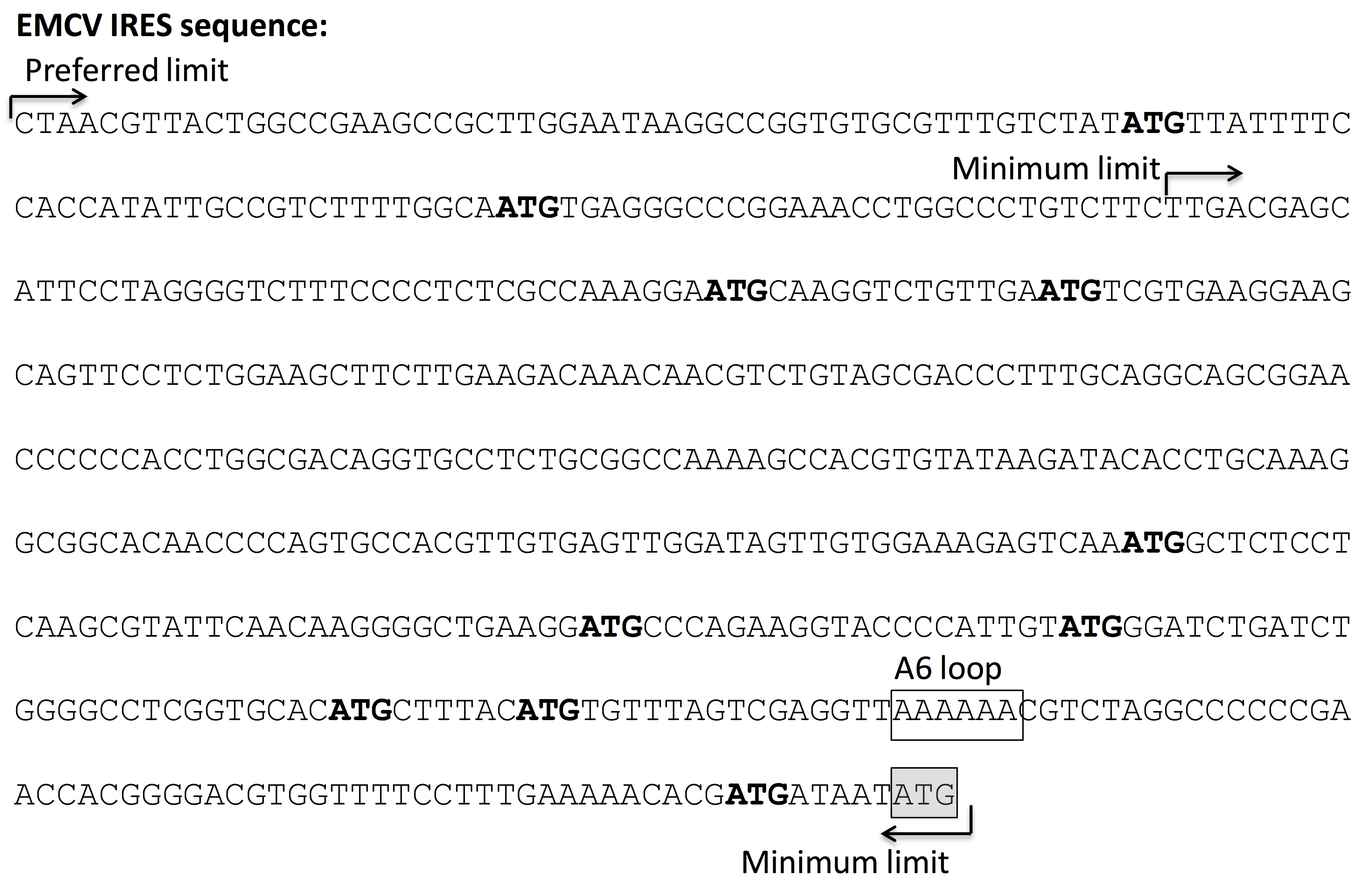

Supplement: S2 Fig — The 557 nucleotide sequence of the EMCV IRES (GenBank Accession: KF836387.1), used in this study to design the B8-IRES-MVA, was confirmed by DNA sequencing, presented here. The optimal EMCV IRES sequence according to Bochkov and Palmenberg [9] should have the A6 loop (boxed) and utilizes the defined native 3’ ATG start codon (in shaded box), not any of the upstream non-defined ATG (in bold). The minimum and preferred boundaries of the EMCV IRES are labelled with arrows. The IRES encompasses the minimum boundaries at both ends, but with the preferred boundary at the 5’ end. (TIFF) [file pone.0127978.s002.tiff]
